# Motion words selectively modulate direction discrimination sensitivity for threshold motion

**DOI:** 10.3389/fnhum.2013.00134

**Published:** 2013-04-10

**Authors:** Andrea Pavan, Māris Skujevskis, Giosuè Baggio

**Affiliations:** ^1^Institut für Psychologie, Universität RegensburgRegensburg, Germany; ^2^Neuroscience Area, SISSA International School for Advanced StudiesTrieste, Italy

**Keywords:** spoken language, discrimination sensitivity, systems interaction, modular theory, embodied theory

## Abstract

Can speech selectively modulate the sensitivity of a sensory system so that, in the presence of a suitable linguistic context, the discrimination of certain perceptual features becomes more or less likely? In this study, participants heard upward or downward motion words followed by a single visual field of random dots moving upwards or downwards. The time interval between the onsets of the auditory and the visual stimuli was varied parametrically. Motion direction could be either discriminable (suprathreshold motion) or non-discriminable (threshold motion). Participants had to judge whether the dots were moving upward or downward. Results show a double dissociation between discrimination sensitivity (d′) and reaction times depending on whether vertical motion was above or at threshold. With suprathreshold motion, responses were faster for congruent directions of words and dots, but sensitivity was equal across conditions. With threshold motion, sensitivity was higher for congruent directions of words and dots, but responses were equally fast across conditions. The observed differences in sensitivity and response times were largest when the dots appeared 450 ms after word onset, that is, consistently with electrophysiology, at the time the up/down semantics of the word had become available. These data suggest that word meanings can alter the balance between signal and noise within the visual system and affect the perception of low-level sensory features.

## Introduction

The human brain is uniquely adapted to instantiate at least two types of representations: symbolic, such as the memories associated with visual or auditory linguistic signs; and perceptual, or the internal states immediately resulting from the stimulation of sensory systems. A recurring question in cognitive science is how these two domains are related in the organization of the mind. Two families of answers have been especially prominent. Modular architectures, originating with Fodor (1983), regard input systems, including speech perception and vision, as informationally encapsulated: a system stores all the information that its computations are going to require, and does not engage general knowledge or information from other systems in processing a domain-specific input. According to the modular theory, symbolic and perceptual systems can only interact off-line via inference or impasse-resolving systems (Cooper and Shallice, 2011). Embodied cognition (Barsalou, 1999), on the other hand, proposes that symbolic representations ultimately recruit, to different extents and through a variety of mechanisms, sensory-motor systems. The version of embodied cognition considered here posits that linguistic stimuli (i.e., auditory words) activate relevant sensory representations in a fast and automatic manner. Other versions of the theory hypothesize that symbol processing flexibly recruits information that has been acquired through sensory-motor experience but that may no longer be represented within brain systems responsible for perception and action (Mahon and Caramazza, 2008; Binder and Desai, 2011). Therefore, a broad spectrum of theories exists between modular and embodied architectures, each positing various forms and degrees of interaction between cognition and perception. One aim of research in this field is to locate within the continuum between embodied and modular theories particular instances of symbol processing that may involve perceptual analysis, or conversely, to find cases in which perceptual processing is shaped by exposure to certain symbolic representations.

**Figure 1:**
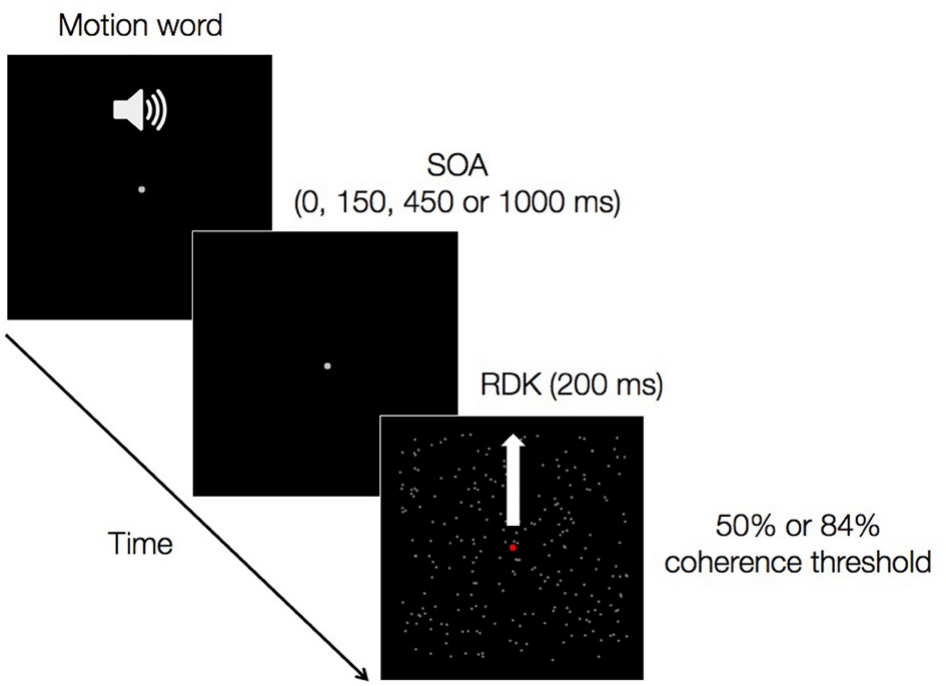
**A trial of the main experiment.** An auditory verb denoting upward or downward motion is followed by a random dot kinematrogram (RDK) presented for 200 ms. The stimulus-onset asynchrony of the auditory and the visual stimuli could be either 0, 150, 450, or 1000 ms. The amount of coherent vertical motion in the RDK was such as to produce either 50 or 84% correct performance in a separate motion discrimination experiment.

This paper focuses on one such case: the influence of language on discriminating invisible visual motion stimuli. Stimuli that do not result in a percept—being constituted by features that in normal conditions would fall below the system's discrimination threshold—seem an appropriate test case for modular and embodied accounts, as will become clear below. In our experiment, each trial consisted of a playback of a spoken verb denoting upward (e.g., “to climb”) or downward (e.g., “to descend”) motion followed by a brief presentation (e.g., 200 ms) of a single visual field of random dots moving upwards or downwards (Figure 1). The meaning of the word could be either congruent or incongruent with the direction of the dots. Participants performed a two-alternative forced-choice (2AFC) task judging whether the dots were moving upward or downward by pressing one of two designated keys. The time interval between the onsets of the auditory and visual stimuli was varied parametrically (stimulus-onset asynchronies, SOAs: 0, 150, 450, and 1000 ms), and the amount of coherent motion in the visual stimuli was set for each participant so as to produce direction-discrimination performance of either 50% or 84% accuracy. SOA values were chosen based on current knowledge of the time course of auditory word recognition and integration (Friederici, 2002): at 150 ms word identity is established; at 450 ms word meaning is fully activated; at 0 and 1000 ms on-line processes are not yet initiated or are already completed, respectively. Below we detail the use of these SOAs in framing our study's hypotheses. These were not concerned with intermediate processing stages between word form identification and semantic activation (e.g., word category identification), therefore we did not include intermediate SOAs between 150 and 450 ms in our design.

Each of the theoretical proposals outlined above makes different predictions for our experiment. According to modular theories, the up/down semantics of the verb cannot influence visual motion discrimination on-line. As a result, in the congruent and incongruent conditions, response times and sensitivity should be equal for the shorter SOA values, regardless of whether motion is at or above threshold. As soon as the full meaning of the verb has been retrieved, between 250 and 550 ms from auditory word onset (Kutas and Hillyard, 1980; Kutas and Federmeier, 2000; Lau et al., 2008; Hagoort et al., 2009), semantic information is broadcast to central systems and may then influence motion discrimination off-line. A matching word could thus speed-up direction-discrimination decisions for suprathreshold motion for longer SOAs. If this is an inference-based process, occurring at the level of central systems, it seems unlikely that its outcome is fed back to sensory systems where it might influence the visual system's sensitivity for discriminating motion at or near threshold. In brief, modularity predicts a difference in response times for suprathreshold motion for longer SOAs, and no effects in the other cases.

Embodied cognition implies that vertical motion words have spatial up/down semantics in virtue of visual motion representations, and these are rapidly recruited as soon as word form is identified. This claim is consistent with recent work suggesting that lexical access can be faster than previously thought. Rapid lexical processing has been reported in a variety of tasks and experimental conditions between ~50 and 120 ms from word onset (Shtyrov and Pulvermüller, 2007; Dell'Acqua et al., 2010; Shtyrov et al., 2010; Kim and Lai, 2012; MacGregor et al., 2012). As a result of this fast activation of word representations—which embodied cognition regards as being sensory in nature—and regardless of whether dot motion direction falls above or below a discrimination threshold, a sensory trace is present in the visual system, affecting the way the direction of the dots is perceived and judged. Accordingly, discrimination responses should be faster in the congruent condition with suprathreshold coherence, similar to the “match advantages” reported in the literature (Zwaan et al., 2004). Moreover, the sensory traces activated by motion verbs may increase discrimination sensitivity for dots moving in that direction if coherence is at threshold. If the activation following word form identification is sensory in nature, the shorter the SOA the more vivid the activated sensory trace, and the stronger its effect on the visual stimulus. Any speed-up effect or increase in sensitivity in the congruent condition is therefore expected to decrease as the SOA increases. In summary, embodied cognition predicts a difference in response times for suprathreshold motion, and a difference in discrimination sensitivity for threshold motion. Both effects should be strongest at the shorter SOAs.

Two studies are relevant for the present experiment. Meteyard et al. (2007) used lists of three types of words—denoting upward, downward, or no motion—with all words within a list being of the same type. Participants heard same-type word lists binaurally at a rate of one word per second. Concurrently, random dot kinematograms (RDKs) showing upward or downward motion at threshold level were presented for 150 ms, separated by a delay of 500–1000 ms. The timing of words and of the visual stimuli was unsynchronized, and each word list of a particular type was presented concurrently with visual motion in a fixed direction. Participants performed a motion-detection task by pressing one key if they saw directional motion, and another key if they saw random motion. Three dependent variables were used: an index of detection sensitivity (d′), a decision criterion, and reaction times. Sensitivity was lower for incongruent relative to congruent trials and controls. The directional/random-motion decision criterion was lower for congruent trials compared to incongruent and control trials. In incongruent trials, relative to congruent and control items, responses were faster for downward motion and slower for upward motion.

The authors acknowledge that such a pattern of effects cannot be easily accounted for by any of the current theories. The sensitivity effect is consistent with embodied cognition as it suggests that incongruent motion words increase noise within the visual system, as though indeed the kind of representation they activate was perceptual in nature. However, the decision criterion effect is consistent with modular theories that predict interference to occur at higher processing stages. As d′s and the decision criterion alone appear to be insufficient to afford a stringent test of modular and embodied accounts, our experiment builds upon the work by Meteyard et al. (2007) by adding different SOAs as a design parameter. As detailed above, embodied cognition predicts changes in sensitivity to be stronger the more recent the sensory trace activated by the word, that is, that the effect will be stronger for some of the smaller time intervals between the auditory and the visual stimulus. In contrast, modular accounts make the opposite prediction, that speech processing and vision interact off-line, a process that is most likely reflected at some of the longer SOAs. Furthermore, our study employs a suprathreshold coherence condition as an additional control, while Meteyard et al. tested motion word interference with coherence at threshold level only. Finally, our experiment adopts a more specific motion discrimination procedure (upward/downward), whereas Meteyard et al. used a motion detection task (directional/random).

In a follow-up study, Meteyard et al. (2008) presented participants again with moving dot fields, this time with parameterized vertical motion coherence, including threshold-level, 30%, 60%, and 90%. In this study the order of the visual stimuli and the words was reversed: random dot fields were followed by written up/down verbs or by control verbs that did not denote motion. Participants had to perform a lexical decision task. With threshold-level coherence, reaction times were slower when the direction of the dots and of the word was incongruent. With suprathreshold motion, there were fewer errors for control verbs than for congruent or incongruent verbs. The authors suggest that threshold-level stimuli activate motion area MT of the human brain, which in these conditions is not under executive control and therefore interferes with the lexical decision. They take the absence of a difference in reaction times as evidence for a lack of interference by suprathreshold motion, which is suppressed, being a task-irrelevant stimulus under executive control. The distribution of error rates with suprathreshold motion is regarded as consistent with the disruption of semantic processing following task-irrelevant motion suppression. However, these conclusions are more applicable to the issue of how cognitive control inhibits, or fails to inhibit, low-level distractors depending on whether they fall above or below certain detection or discrimination thresholds (Tsushima et al., 2006), but appear less relevant for testing theories of relationships between semantics and perceptual processing. Indeed, the data by Meteyard et al. (2007, 2008) provide no evidence that, as would be required by the embodied theory, the representations activated by words are sensory in nature, or that, as in certain weaker versions of the theory, interplay between words and visual stimuli occurs at the level of sensory systems. In our study, we attempted to obtain (counter-)evidence on these issues using different SOAs and different threshold levels, whereas no-motion control words are dropped to reduce total running time, and on the grounds that neither of the theories considered here makes predictions that require a distinction between facilitation and interference effects.

## Methods

### Apparatus

Stimuli were generated with Psychtoolbox for Matlab (Brainard, 1997; Pelli, 1997) and displayed on a 22-inch LCD SAMSUNG Syncmaster 2233RZ monitor with a refresh rate of 120 Hz (frame duration: 8.33 ms) (Wang and Nikolić, 2011). The screen resolution was 1680 × 1050 pixels. Each pixel subtended ~1.7 arcmin. The minimum and maximum luminance of the screen were 0.19 and 134 cd/m^2^, respectively. Screen luminance was measured with a Minolta LS-100 photometer. A gamma-corrected lookup table (LUT) was used to ensure that luminance was a linear function of the digital representation of the image.

### Participants

Participants were 14 Italian native speakers (7 female; age *M* = 25.07, *SD* = 2.23) with normal or corrected-to-normal visual acuity. Viewing was binocular and hearing was binaural. Participants had enrolled voluntarily for monetary compensation, and had given their informed consent prior to each session.

### Auditory stimuli

The linguistic materials were 40 infinitive verbs in Italian: 20 denoted upward motion (e.g., “salire”) and 20 downward motion (e.g., “scendere”). Word form frequency was matched between up and down verbs using the CORIS/CODIS corpus (Rossini Favretti et al., 2002). Mean word length in letters was 8.6 (*SD* = 1.76) for upward verbs and 8.85 (*SD* = 1.35) for downward verbs (Welch's two-sample *t*-test; *p* > 0.1).

Verbs were read by a male speaker instructed to maintain his reading pace and pitch constant during the recording. The resulting samples (16-bit mono, 44.1 kHz rate) were denoised based on a representative background noise profile, DC offset was removed, and maximum amplitudes were normalized to -3 dB. Audio samples were cut at about 25 ms before the onset of the speech waveform, and at about 50 ms after waveform offset. Therefore, the onset of the auditory stimulus, as used in defining SOAs, precedes by about 25 ms the actual speech onset. The mean duration of the resulting traces was 1.17 s for upward motion words (*SD* = 0.2) and 1.21 s for downward motion words (*SD* = 0.14).

### Visual stimuli

Visual stimuli were RDKs made up by 250 white dots (dot diameter: 0.03°) presented within a square aperture of 8° × 8° (density: 3.9 dots/deg^2^) at the center of the screen. The luminance of all dots was set to 66.24 cd/m^2^. The dots were moving against a black background (0.19 cd/m^2^) (Michelson contrast: 0.99) with a speed of 20.1°/s. Dots had a limited lifetime: after 83.3 ms each dot vanished and was replaced by a new dot at a different, randomly selected position within the square window. In the motion sequence each dot had equal probability of being selected as a signal dot (Newsome and Paré, 1988; Stevens et al., 2009). In addition, at the beginning of each trial an “age” value (ranging from 8.3 to 200 ms) was assigned to each dot: a dot could appear on the first frame or sometime within a temporal window ranging from the first frame to 200 ms; in each trial dots appeared asynchronously on the display. This choice was implemented to minimize the presence of local “motion streaks” (Geisler, 1999) that could provide cues for direction discrimination. In addition, moving dots that traveled outside the window were replaced by a new dot at a different random location within the circular window, thus maintaining a constant dot density throughout the stimulus presentation. Dots were either constrained to move along upward/downward translational trajectories (signal dots) or were positioned in new locations, randomly selected within the circular window, on each successive frame of the sequence (noise dots) (Scase et al., 1996).

Each RDK consisted of a 24-frame motion sequence (200 ms) in which a certain percentage of dots were signal dots, whereas the remaining dots were noise. We employed such stimulus duration to prevent covert attentional tracking of the stimuli (Martinez-Conde et al., 2004; Wright and Ward, 2008). The spatiotemporal characteristics of the RDKs matched those reported in a previous investigation on global motion and ensured that false matches across successive frames were negligible and that the correspondence problem was minimized (Williams and Sekuler, 1984; Simmers et al., 2003; Stevens et al., 2009).

### Procedure

#### Step 1: coherence thresholds estimates

Prior to the main cross-modal direction-discrimination task (step 3 below), observers performed a preliminary direction-discrimination task in order to estimate subject-specific motion coherence thresholds (CTs). Participants sat in a dark sound-attenuated room 57 cm away from the screen. A chin rest was used to maintain head position. Observers had to fixate a central red fixation point (diameter: 0.4°) for 1000 ms. After the fixation point had disappeared, a RDK was displayed at the center of the screen for 200 ms. The RDK contained a certain percentage of signal dots, and the remaining dots were noise. Signal dots drifted either upwards or downwards.

A 2AFC procedure was employed to estimate the observers' CTs, corresponding to 50% (threshold motion) and 84% (suprathreshold motion) correct responses. The two motion coherence levels were estimated in separate runs whose order was randomized across participants. The percentage of coherently moving dots (signal dots) was varied by manipulating the relative percentage of signal and noise dots using a simple up-down staircase in the case of 50% CT and a 1 up-4 down staircase for 84% CT (Levitt, 1971). The staircase started at a random value between 15 and 30% of motion coherence, and terminated after either 100 trials or 16 reversals. The initial step size of the staircase was set at 10% of coherently moving dots, then after each reversal the step size was decreased until a minimum value of 0.1% was reached. The motion direction of the RDKs was randomized across trials with the constraint that the same direction could not be presented in more than three consecutive trials. Observers had to indicate the direction of signal dots in the target RDK by pressing one of two designated keys on a standard keyboard. At the end of the procedure, the threshold was calculated by averaging the modulation values of the last 12 reversals.

#### Step 2: direction-discrimination controls

Prior to the main experiment (step 3 below), participants performed a 2AFC direction-discrimination task aimed to assess whether the CTs estimated in step 1 indeed produced ~50 and 84% correct responses. The exact same direction-discrimination task was performed also after the main experiment to control for possible perceptual learning effects that could have occurred during the main task. RDKs were presented at the center of the screen for 200 ms. The CTs estimated in the previous step of the procedure were used to set participant-specific coherence values and to test whether these would produce the expected accuracies (~50 and 84%). The task consisted of 20 trials with motion direction randomized across trials and with the constraint that the same motion direction could not be presented in more than three consecutive trials.

#### Step 3: cross-modal direction discrimination

In the main task, participants were instructed to fixate a 1000 ms white point (diameter: 0.4°) displayed at the center of the screen. Then an upward or downward motion word was presented. Finally, the RDK was shown for 200 ms. The time interval between the onset of the auditory word and the onset of the RDK (i.e., SOA) could be either 0 or 150 or 450 or 1000 ms (Figure 1). From the visual onset of the RDK participants had 2000 ms to judge its motion direction in 2AFC task. Participants were instructed to be as fast and accurate as possible. CTs corresponding to 50 and 84% accuracy were presented in separate blocks. Each block consisted of 160 trials. That is, each motion word was repeated four times (i.e., for each SOA level) giving rise to 80 upward and 80 downward trials. Initially, the motion direction of the RDK was balanced over trials ensuring that, for instance, for the 80 upward motion word trials, 40 were paired with a RDK moving upward (match condition) and 40 with a RDK moving downward (mismatch condition). The same applied to the 80 downward word trials. Subsequently, motion direction, words, and SOA levels were randomized across trials, with the constraint that the same motion direction could not be presented in more than three consecutive trials. The experimental procedure took ~2.5 h to be completed.

### Data analysis

Mean values across trials and across subjects with corresponding standard errors of mean (SEM) were computed in each condition for reaction times, d′s and a measure of the internal response criterion or bias (C). A natural log (ln) transformation of reaction times of correct responses was performed to lessen the influence of outliers (Fazio, 1990).

Response accuracy cannot be used as an indicator of the degree to which direction discrimination of visual motion was modulated by the word semantics as responses are likely to be biased in the direction suggested by the word, regardless of whether visual motion was actually perceived. For this reason, we computed d′s and the criterion or bias C for 2AFC tasks with observer bias (Kingdom and Prins, 2010), classifying the observer's responses as “hits” or “false alarms.” For example, an upward response was designated as a hit if the RDK was moving upward and as a false alarm if the RDK was moving downward. The d′s and C were computed using MATLAB Palamedes (Prins and Kingdom, 2009) as follows:

(1) $$d^{\prime }=\frac{z(pH)-z(pF)}{\sqrt{2}}$$

(2) $$C=\frac{-[z(pH)+z(pF)]}{\sqrt{2}}$$

where *z(pH)* and *z(pF)* are the *z*-value of the probability of “hits” and “false alarms,” respectively. To increase statistical power, we collapsed data from the two motion directions within each condition (congruent/incongruent).

We performed separate Two-Way repeated measures ANOVA for threshold and suprathreshold experimental blocks (i.e., for 50 and 84% correct performance) with the factors Condition (with 2 levels: the direction of the word and RDK is congruent or incongruent) and SOA (4 levels: 0, 150, 450, and 1000 ms), and using d′s, C and ln-transformed reaction times as dependent variables. The results of ANOVA are reported in Table 1. Mauchly's test was carried out to assess the sphericity of the data, but the results never achieved significance. Reaction times, d′s and C were further compared across conditions at particular SOA values, providing a more fine grained statistical picture than that afforded by ANOVA. Some of our data series were not normally distributed, as assessed by Shapiro-Wilk normality tests, hence we employed non-parametric Wilcoxon signed rank tests instead of *t*-tests, comparing d′s, C or ln-transformed reaction times across the congruent and incongruent conditions at the relevant SOA values. The results of these additional statistical tests are reported in Table 2.

**Table 1:** **Summary of results of ANOVA statistics for the main cross-modal direction-discrimination experiment**.

	**Congruency**	**SOA**	**Congruency × SOA**
log RTs 50%	*F*_(1, 13)_ = 0.064	*F*_(1, 13)_ = 3.555	*F*_(3, 39)_ = 0.15
	*P* = 0.805	*p* = 0.0229^*^	*p* = 0.929
log RTs 84%	*F*_(1, 13)_ = 5.334	*F*_(1, 13)_ = 1.839	*F*_(3, 39)_ = 1.207
	*p* = 0.038^*^	*p* = 0.156	*p* = 0.32
d′ 50%	*F*_(1, 13)_ = 8.205	*F*_(1, 13)_ = 0.321	*F*_(3, 39)_ = 1.412
	*p* = 0.0133^*^	*p* = 0.81	*p* = 0.254
d′ 84%	*F*_(1, 13)_ = 3.804	*F*_(1, 13)_ = 1.685	*F*_(3, 39)_ = 1.347
	*p* = 0.073	*p* = 0.186	*p* = 0.273
Criterion 50%	*F*_(1, 13)_ = 0.048	*F*_(1, 13)_ = 2.11	*F*_(3, 39)_ = 1.968
	*p* = 0.83	*p* = 0.115	*p* = 0.135
Criterion 84%	*F*_(1, 13)_ = 0	*F*_(1, 13)_ = 2.311	*F*_(3, 39)_ = 3.105
	*p* = 0.983	*p* = 0.0913	*p* = 0.0374^*^

Significant effects are indicated with an asterisk (^*^).

**Table 2:** **Summary of results of Wilcoxon signed rank tests for the main cross-modal direction-discrimination experiment**.

	**0 ms**	**150 ms**	**450 ms**	**1000 ms**
d′ 50%	*V* = 89	*V* = 68.5	*V* = 95	*V* = 46
	*p* = 0.02	*p* = 0.33	*p* = 0.005^*^	*p* = 1
log RTs 84%	*V* = 35	*V* = 45	*V* = 12	*V* = 38
	*p* = 0.296	*p* = 0.67	*p* = 0.009^*^	*p* = 0.391

Bonferroni-corrected alpha is 0.0125. Significant effects are indicated with an asterisk (^*^).

## Results

### Coherence thresholds and pre-/post-accuracies

The mean CT for 50% correct responses was 2.42% (*SE* = 0.57) of moving dots, whereas the CT for 84% correct responses was 8.48% (*SE* = 1.66). The mean accuracies in the direction-discrimination task (Step 2) using CTs estimated for 50% correct responses (threshold-level coherence) were 0.51 (*SE* = 0.022) and 0.55 (*SE* = 0.017) before and after the main experiment respectively. A one-sample *t*-test relative to chance level shows a significant difference in the post-experiment discrimination task [*t*_(13)_ = 2.95, *p* = 0.011], suggesting a weak perceptual learning effect during the main experiment. The mean accuracies obtained in the pre- and post-direction-discrimination tasks using the CT estimated for 84% correct responses (suprathreshold coherence) were 0.80 in both cases (*SE* = 0.02 and 0.03, respectively), and did not significantly differ from 0.84 [*t*_(13)_ = −1.75, *p* = 0.1 and *t*_(13)_ = −1.22, *p* = 0.24, respectively for the pre- and post-experiment discrimination tasks].

### Cross-modal direction discrimination and reaction times

**Figure 2:**
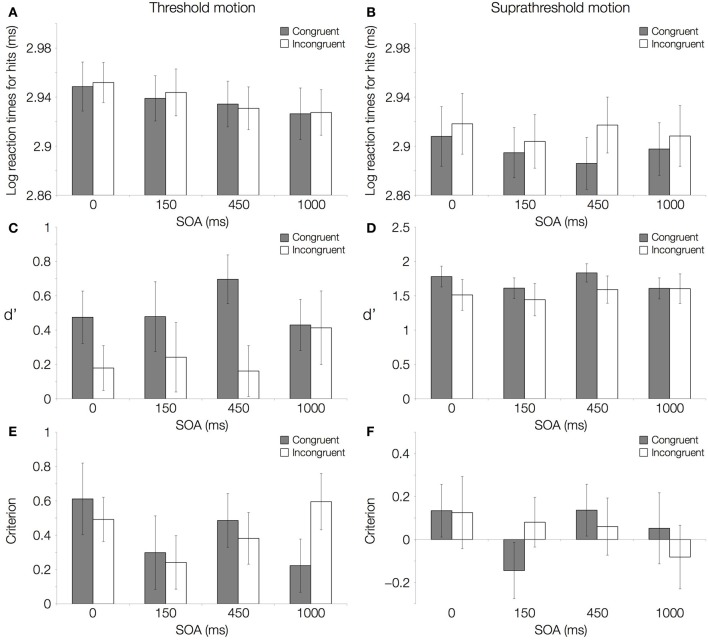
**Results of the main experiment.** Log transformed reaction times (ms), direction-discrimination sensitivity (d′), and criterion with motion coherence at threshold level (50% correct performance) and at suprathreshold level (84% correct performance) are shown. **(A)** Log transformed reaction times for hits with motion coherence at threshold level and **(B)** at suprathreshold level. **(C)** Direction-discrimination sensitivity (d′) with motion coherence at threshold level and **(D)** at suprathreshold level. **(E)** Criterion with motion coherence at threshold level and **(F)** at suprathreshold level. All panels show a comparison of congruent (gray; matching directions of word and RDK) and incongruent (white; mismatching directions of word and RDK) conditions.

The results of the main experiment show a double dissociation between response times and direction-discrimination sensitivity (d′) depending on motion CTs. No reaction time difference was observed between congruent and incongruent trials with 50% threshold-level motion (Figure 2A; Table 1). Responses became faster as the interval between the onsets of the word and the visual stimulus increased. Despite responses being equally fast in the two conditions, direction-discrimination sensitivity was higher for congruent relative to incongruent trials (Figure 2C; Table 1), and this difference in discrimination sensitivity (d′) was largest when the visual stimulus was shown 450 ms after word onset (Table 2).

With 84% suprathreshold motion, the opposite pattern of effects was found. Responses were faster with congruent word and visual dots directions (Figure 2B; Table 1), and the largest difference in reaction times was observed for the 450 ms SOA value (Table 2). Direction-discrimination sensitivity was equal in the congruent and incongruent conditions (Figure 2D; Table 1).

There were no differences for the criterion or bias (C) between congruent and incongruent trials (Figures 2E,F; Table 1), suggesting that the observed sensitivity effects cannot be accounted for by differences across conditions in the extent to which auditory words bias responses to visual stimuli. An interaction between Congruency and SOA for suprathreshold stimuli was found (Table 1). Crucially, however, for the SOA at which the effects in d′ and reaction times were largest (450 ms) the criterion did not differ from zero in either the congruent or the incongruent conditions, for threshold or suprathreshold stimuli (one-sample Wilcoxon signed rank tests, *p* > 0.0125 Bonferroni-corrected).

## Discussion

The present study was aimed at testing predictions from modular and embodied accounts of the relations between spoken language and visual perception. Auditory up/down motion words were followed, after time intervals of varying length, by visual fields containing random dots moving either upward or downward with two levels of coherent motion, such that the direction of the dots was either discriminable (above threshold motion) or non-discriminable (threshold motion). We found a double dissociation between reaction times and direction-discrimination sensitivity depending on whether dot motion was at or above threshold. With threshold motion responses were equally fast, but sensitivity was higher when the direction of the word and dots matched. With above threshold motion sensitivity to the direction of dots was equal across conditions, but responses were faster with matching directions of word and dots. The observed difference in sensitivity at threshold as well as the difference in reaction times above threshold were largest when the visual stimulus was presented 450 ms after word onset.

Modular theories predict that lexical semantics can only influence visual motion discrimination off-line. Thus, a difference in response times should be observed at the longer SOAs with suprathreshold motion. Our results are only partly consistent with this prediction in that responses were faster with congruent directions of word and dots at 450 ms but not at 1000 ms SOA. If interaction occurred off-line, when automatic processes within each system are completed, there should be an effect at 1000 ms SOA too. Our data suggest instead that on-line linguistic processes, arguably at some stage of lexico-semantic activation (see below), determine a more specific time window for systems interaction. Moreover, modular theories consider the relation between informationally encapsulated systems to be mediated by inference. However, it seems difficult to produce a model of how exactly inference could alter perceptual sensitivity in a task such as the present one. Our finding that direction-discrimination sensitivity is selectively modulated by congruency of the word and dots seems at odds with the modular notion that input systems interact via inference.

Embodied cognition predicts that motion words activate sensory traces within the visual system, affecting how dot motion is perceived and judged. With motion above threshold, faster responses in congruent trials could be accommodated within the theory as a “match advantage” (Zwaan et al., 2004), that is, a speed-up effect produced by the congruency between the perceptual features activated by the word and the subsequent visual stimulus. If words immediately activate sensory representations, the closer in time the onset of the word and the dots, the stronger the response time speed-up. However, we found no reaction time differences for the shorter SOAs, and an effect was observed only at 450 ms SOA, suggesting that interaction can occur only at specific stages of lexical processing (see below). Similarly, any difference in sensitivity at threshold should be more marked when the time interval between word and dots is shorter. However, the strongest modulation of discrimination sensitivity at threshold was again found at 450 ms.

Although there exist different views and requirements for modular (Fodor, 1983; Bever, 1992; Coltheart, 1999; Cooper and Shallice, 2011) and embodied theories (Barsalou, 1999; Mahon and Caramazza, 2008; Binder and Desai, 2011), our results are not entirely consistent with the core modular or the core embodied hypothesis. Our data do not support the view that input systems are informationally encapsulated, that is, unable to interact on-line and unlikely to display central-to-peripheral modulations of perceptual sensitivity, nor do our results agree with the notion that representations associated with words are sensory in nature, and thus liable to produce the largest effects on perceptual sensitivity in a rapid and automatic fashion.

Our findings are compatible with a broad range of experimental results on interactions between visual motion perception and processing in other sensory systems. Stein et al. (1996) observed that an auditory cue increased the perceived intensity of a visual stimulus, and Vroomen and de Gelder (2000) showed that a high tone embedded in a sequence of low tones increased the detection of visual targets. Sekuler et al. (1997) found that two disks moving to converge on the same point in space to then travel apart were perceived as bouncing off each other if a sound was presented at the point of visual coincidence. If there was no sound, participants perceived the disks as continuing in their original direction. McDonald et al. (2000) showed that a sudden sound can improve the detection of a subsequent flash at the same location in space. These studies suggest that interplay between visual and auditory systems can occur at lower processing levels than those investigated here, and with less specific and less information-rich auditory stimuli: the sounds that were employed acquired their function as cues only by appearing at a certain time during the presentation of the visual stimulus, or by being part of a stimulus sequence with certain temporal properties.

Similar interaction effects were found when the sound stimuli did carry some motion information. Aspell et al. (2000) presented participants with trials beginning with a noise phase, during which the sound stimulus was stationary and the visual dots moved at random, followed by a coherent phase, in which a percentage of the dots moved coherently either leftward or rightward. Simultaneously, the sound moved in the same or in the opposite direction. Participants had to report either the direction of the dots or the direction of the sound. The concurrent visual motion enhanced performance on the auditory task, and conflicting visual motion degraded performance. Meyer and Wuerger (2001) also used simultaneous visual and auditory stimuli, and found that supratreshold auditory motion biases the perceived visual motion in the direction of the auditory motion, although other studies revealed that the increase in sensitivity can also be direction-blind. Similar results were obtained for consistent and inconsistent motion conditions (Wuerger et al., 2003; Patching and Quinlan, 2004).

Our experiment extends results from this line of research by showing that auditory stimuli carrying abstract motion information (i.e., verbs with motion semantics) can bias response times and discrimination sensitivity in ways compatible with previous studies. We found a double dissociation of response times and discrimination sensitivity depending on whether motion is at or above threshold, suggesting the existence of a complex mechanism underlying the integration of visual and auditory motion information. One possibility is that a form of on-line cross-modal compensation allows a perceptual system (vision) to harness information from other brain systems (linguistic semantics) and gain sufficient evidence on the stimulus to attain the desired behavioral goal. If, however, the unimodal (visual) input stream provides adequate sensory evidence, the perceptual system may operate in a more autonomous manner, thus increasing the overall processing speed. On this view, the particular degree of informational encapsulation displayed by a system may depend on the richness and variety of information available in the current perceptual setting as well as on task demands. This theoretical sketch may explain both the modulation in sensitivity for threshold motion (the visual system attempts to compensate for the lack of sensory evidence, given the low motion coherence within the visual stimulus, harnessing word information in order to successfully perform the discrimination task) and the difference in response times for suprathreshold motion (as a result of an increase in processing speed afforded by the coherent and unambiguously directional visual signal). Any conclusion must at this stage remain tentative and serve as a starting point for testing on-line cross-modal compensation as a middle ground between modular and embodied theories of mind.

Perhaps most significantly, we found sensitivity and response times to differ most across conditions when the onsets of the visual dots and of the auditory word were 450 ms apart. Combined with the electrophysiological finding that lexical meanings become available for contextual integration between 250 and 550 ms from word onset (Kutas and Hillyard, 1980; Kutas and Federmeier, 2000; Lau et al., 2008; Hagoort et al., 2009; for more precise estimates of the timing of semantic access, see Baggio, 2012; Baggio and Fonseca, 2012), our data suggest that the strongest cross-modal interactions occur within the time interval in which the up/down semantics of the word is active. Supporting evidence for this conclusion comes from an fMRI study (Meyer et al., 2011) in which incongruent combinations of meaningful speech and body actions produced increased activation of the cortical network underlying semantic processing, including the bilateral posterior superior temporal sulcus (pSTS) and the left inferior frontal gyrus, but not in premotor cortex, which supports instead the integration of sensory information (motion direction) instead (Wuerger et al., 2012). If this account is correct, our study suggests that the integration of visual information and spoken language occurs at the semantic level, and not at the level of perceptual systems as predicted by embodied cognition.

### Conflict of interest statement

The authors declare that the research was conducted in the absence of any commercial or financial relationships that could be construed as a potential conflict of interest.
